# Estimation of the overall burden of cancers, precancerous lesions, and genital warts attributable to 9-valent HPV vaccine types in women and men in Europe

**DOI:** 10.1186/s13027-017-0129-6

**Published:** 2017-04-11

**Authors:** Susanne Hartwig, Jean Lacau St Guily, Géraldine Dominiak-Felden, Laia Alemany, Silvia de Sanjosé

**Affiliations:** 1 Department of Epidemiology, Sanofi Pasteur MSD, 162 avenue Jean Jaurès, Lyon, France; 2 Department of Otolaryngology-Head and Neck Surgery, Tenon Hospital – Assistance Publique-Hopitaux de Paris (AP-HP) and Sorbonne University-Paris 6, Pierre-et-Marie Curie University Cancerology Institute, 4 rue de la Chine, 75020 Paris, France; 3 Cancer Epidemiology Research Program, Institut Català d’Oncologia (ICO)-IDIBELL, L’Hospitalet de Llobregat, Catalonia Spain; 4 CIBER Epidemiologia y Salud Pública, Barcelona, Spain

**Keywords:** Human papillomavirus, Burden of disease, Cancer, Precancerous lesions, Genital warts, Head and neck

## Abstract

**Background:**

In addition to cervical cancer, human papillomavirus (HPV) is responsible for a significant proportion of cancers and precancerous lesions of the vulva, vagina, anus, penis, head and neck, as well as genital warts. We estimated the annual number of new cases of these diseases attributable to 9-valent HPV vaccine types in women and men in Europe.

**Methods:**

The annual number of new cancers of the cervix, vulva, vagina, anus, penis, and selected head and neck sites in the population of the European Medicines Agency territory was estimated based on age-specific incidence rates extracted from Cancer Incidence in 5 Continents, Volume X and Eurostat population data for 2015. The annual number of new cancers attributable to 9-valent HPV vaccine types was estimated by applying the HPV attributable fraction from reference publications based on a large European multicenter study. For non-cervical cancers, HPV attributable fractions were based on oncogenically-active HPV infections only (i.e., detection of HPV DNA and either mRNA and/or p16 positivity). For precancerous lesions of the cervix, vulva, vagina, and anus, and for genital warts, previously published estimations were updated for the 2015 population.

**Results:**

The annual number of new cancers attributable to 9-valent HPV vaccine types was estimated at 47,992 (95% bound: 39,785-58,511). Cervical cancer showed the highest burden (31,130 cases), followed by head and neck cancer (6,786 cases), anal cancer (6,137 cases), vulvar cancer (1,466 cases), vaginal cancer (1,360 cases), and penile cancer (1,113 cases). About 81% were estimated to occur in women and 19% in men. The annual number of new precancerous lesions (CIN2+, VIN2/3, VaIN2/3, and AIN2/3) and genital warts attributable to 9-valent HPV vaccine types was estimated at 232,103 to 442,347 and 680,344 to 844,391, respectively.

**Conclusions:**

The burden of cancers associated with 9-valent HPV vaccine types in Europe is substantial in both sexes. Head and neck cancers constitute a heavy burden, particularly in men. Overall, about 90% of HPV-related cancers, 80% of precancerous lesions, and 90% of genital warts are expected to be attributable to 9-valent HPV vaccine types each year, demonstrating the important preventive potential of the 9-valent HPV vaccine in Europe.

**Electronic supplementary material:**

The online version of this article (doi:10.1186/s13027-017-0129-6) contains supplementary material, which is available to authorized users.

## Background

Human papillomavirus (HPV) infections are the most common sexually transmitted infections, with more than 40 HPV types that can be transmitted through direct sexual contact, of which about a dozen are classified as high-risk types. Persistent infection with high-risk HPV types can cause cellular changes that may progress to cancer or precancerous lesions of the cervix, vagina, vulva, anus, penis, and head and neck. On the other hand, low-risk types can cause genital warts and recurrent respiratory papillomatosis (RRP), but cause cancer very rarely [1]. Indeed, the high-risk HPV types 16, 18, 31, 33, 45, 52, and 58 are responsible for about 90% of cervical cancers worldwide, whereas HPV6 and 11 are low-risk types for cancer but are responsible for about 90% of warts on or around the genitals, anus, mouth, and throat (RRP) [2].

The quadrivalent HPV vaccine, Gardasil®/Silgard® (Sanofi Pasteur MSD/Merck Sharp & Dohme), protects against infection with HPV16, 18, 6, and 11, and was licensed in Europe in 2006. It was followed in 2007 by the bivalent HPV vaccine, Cervarix® (GlaxoSmithKline Biologicals), which protects against infection with HPV16 and 18. Both vaccines have reassuring safety profiles, as demonstrated in clinical trials and several large post-licensure studies [3], and they provide a high level of protection against HPV16- and 18-attributable lesions.

In 2015, the 9-valent HPV vaccine, Gardasil9 (Sanofi Pasteur MSD/Merck Sharp & Dohme), was licensed in Europe for the prevention of cancers and precancerous lesions of the cervix, vulva, vagina, and anus, as well as genital warts caused by HPV6, 11, 16, 18, 31, 33, 45, 52, and 58 [4]. This vaccine protects against five high-risk HPV types not included in first-generation HPV vaccines (HPV31, 33, 45, 52, and 58) [1].

We recently published an estimation of the annual burden of cancers and precancerous lesions of the cervix, vulva, vagina, and anus, and of genital warts attributable to 9-valent HPV vaccine types in Europe in 2013, and compared it to the estimated annual burden of the same lesions attributable to quadrivalent HPV vaccine types [5]. Recently, two new papers [6, 7] based on a large European multicenter study have published information on HPV type distribution and HPV attributable fractions in head and neck and penile cancers, which gave us the opportunity to provide robust estimates on the contribution of 9-valent HPV vaccine types at these sites as well. Therefore, in order to provide a more exhaustive overview of the preventive potential of the 9-valent HPV vaccine, we have updated our previous estimates to 2015. These new estimates include the annual number of new cancers of the cervix, vulva, vagina, anus, penis, and head and neck; the annual number of new precancerous lesions of the cervix, vulva, vagina, and anus; and the annual number of genital warts cases attributable to 9-valent HPV vaccine types, including the low-risk types HPV6 and 11, in Europe in 2015.

## Methods

### Estimation of the annual number of new HPV-related cancers in Europe

The present evaluation was based on cancer incidence data from Cancer Incidence in Five Continents (CI5) Volume X, which were collected from 2003 through 2007. The CI5 database is available on the website of the International Agency for Research on Cancer (IARC) [8] and contains worldwide data on cancer incidence rates classified by International Classification of Diseases 10^th^ Revision (ICD-10) codes. These data are obtained from regional or national registries, depending on the country, but to be included in CI5 these registries must meet the IARC’s quality criteria, i.e., they must have reliable cancer registry data. We compiled data on 32 countries: 31 countries covered by the European Medicines Agency (EMA, Austria, Belgium, Bulgaria, Croatia, Cyprus, the Czech Republic, Denmark, Estonia, Finland, France, Germany, Greece, Hungary, Iceland, Ireland, Italy, Latvia, Liechtenstein, Lithuania, Luxemburg, Malta, the Netherlands, Norway, Poland, Portugal, Romania, Slovenia, Slovakia, Spain, Sweden, and the United Kingdom) and one not covered by the EMA (Switzerland).

The information in CI5 Volume X was obtained from national cancer registries for Belgium, Bulgaria, Croatia, Cyprus, the Czech Republic, Denmark, Estonia, Finland, Iceland, Ireland, Latvia, Lithuania, Malta, the Netherlands, Norway, Slovenia, Slovakia, and Sweden; and from regional cancer registries for Austria, France, Germany, Italy, Poland, Portugal, Spain, Switzerland, and the United Kingdom. To ensure that national populations were adequately represented in countries where only regional cancer registries exist, we assessed the geographical coverage and distribution of these registries.

Five of the 32 countries selected did not have data available in CI5 Volume X: Greece, Hungary, Liechtenstein, Luxemburg, and Romania. Because of its very low population size, we excluded Lichtenstein from our analysis. For the remaining four countries, we extrapolated age-specific average cancer incidence rates from neighboring countries, or from cancer registries in the same area as the countries selected. The choice of countries used for extrapolation was the same as that used for Globocan [9]. Thus, for Greece data from Bulgaria, Cyprus, and Central Serbia were used; for Hungary data from Austria, Croatia, Central Serbia, Slovakia, and Slovenia were used; for Luxemburg data from French and German cancer registries were used; and for Romania data from Bulgaria, Slovakia, and one regional registry in Romania were used. In conclusion, our results refer to a geographical region of 31 European countries.

The incidence of cancer of the cervix (ICD-10 code C53), vulva (C51), vagina (C52), anus (C21), penis (C60), and selected head and neck cancers that are known to be at least partially related to HPV [6]: oral cavity cancers (C02-06), nasopharyngeal cancers (C11), oropharyngeal cancers (C01, C09, C10), hypopharyngeal cancers (C12-13), unspecified pharyngeal cancers (C14), and laryngeal cancers (C32) [6] was derived from CI5 Volume X. Based on these data, we estimated the mean annual number of new cancers at these sites in 2015 in the 31 selected countries by extrapolating the sex- and age-specific cancer incidence data [10] to the population of each country, using 2015 Eurostat population data [11]. Calculations were performed as follows:

$$ \begin{array}{l} Total\  nb\  of\  new\  cases\\ {}=\frac{{\displaystyle {\sum}_{Countries}}\left\{{\displaystyle {\sum}_{age=0}^{85+}}\left( AIR\  in\  male\ast population+{\displaystyle {\sum}_{type\  of\  cancer}}\left( AIR\  in\  female\ast population\right)\right)\right\}}{100,000}\end{array} $$

where AIR is the age- and sex-specific annual incidence rate, and population is the age- and sex-specific population of a given country. The estimated number of new cancers at these sites in all the selected countries were then summed to obtain the overall European burden of these cancers.

### Estimation of the annual number of new cancers attributable to all HPV types and to 9-valent HPV vaccine types in Europe

The number of new cancers attributable to all HPV types and to 9-valent HPV vaccine types (HPV6, 11, 16, 18, 31, 33, 45, 52, and 58) was estimated by applying the corresponding cancer site-specific HPV attributable fraction. For cervical cancers, the HPV attributable fraction was considered to be 100%, as it is generally accepted that HPV infection is necessary for the development of cervical cancer [12]. However, for all other cancer sites, the fraction attributable to all HPV types and to 9-valent HPV vaccine types was based on the prevalence of oncogenically-active HPV infections (i.e., detection of HPV DNA and either mRNA and/or p16 positivity), which was taken from reference publications based on a large European multicenter study (Table 1). These publications contain the most relevant cancer site-specific data published to-date [6, 7, 13–16] and covered all our selected sites. European data on oncogenically-active HPV infections were not directly available in these publications; they were obtained from the authors, who are also co-authors of the present study (LA and SdS). To avoid overestimating the contribution of individual HPV types due to multiple infections, the contribution of individual HPV types to multiple infections was calculated under a weighting attribution, proportional to the prevalence of each individual HPV type in single infections. We always used European-specific crude data and the same contribution estimates were used for both sexes.

**Table 1:** Fraction of cancers attributable to all HPV types and to 9-valent HPV vaccine types^a^ by cancer site

Cancer site	ICD-10 code	Fraction attributable to all HPV types % [95% CI]^b^	Fraction attributable to 4-valent HPV vaccine types (6/11/16/18) % [95% CI]^b^	Fraction attributable to 9-valent HPV vaccine types (6/11/16/18/31/33/45/52/58) % [95% CI]^b^	Reference
Cervix	C53	100^c^	72.9 [71.0-74.8]	89.1 [87.7-90.4]	[13]
Vulva	C51	15.9 [13.5-18.4]	84.4 [77.3-90.0]	94.3 [89.1-97.5]	[14]
Vagina	C52	70.2 [62.2-77.4]	72.6 [63.1-80.9]	87.1 [78.8-92.6]	[16]
Anus	C21	87.1 [81.0-91.8]	91.5 [85.7-95.6]	94.4 [89.2-97.5]	[15]
Penis	C60	29.0 [24.7-33.7]	78.9 [70.6-85.9]	90.7 [84.1-95.3]	[7]
Oral cavity	C02-06	3.7 [2.4-5.6]	90.9 [70.8-98.9]	90.9 [70.8-98.9]	[6]
Nasopharynx	C11	10.8 [3.0-25.4]	75.0 [19.4-99.4]	75.0 [19.4-99.4]	[6]
Oropharynx	C01, C09, C10	19.9 [17.2-22.8]	93.8 [88.8-97.0]	97.5 [93.7-99.3]	[6]
Hypopharynx	C12-13	2.4 [0.3-8.4]	50.0 [1.3-98.7]	100 [15.8-100]	[6]
Pharynx	C14	25.0 [10.7-44.9]	85.7 [42.1-99.6]	85.7 [42.1-99.6]	[6]
Larynx	C32	2.4 [1.2-4.1]	66.7 [34.9-90.1]	91.7 [61.5-99.8]	[6]

*CI* confidence interval, *HPV* human papillomavirus, *ICD-10* International Classification of Diseases, 10^th^ Revision, *CI* confidence interval

^a^Adjusted for multiple infections; ^b^Except for cervical cancer, prevalence is based on oncogenically-active HPV infections only (i.e., HPV DNA detection plus either E6*I mRNA expression or p16 overexpression). ^c^HPV is the necessary cause for cervical cancer [12]

### Estimation of the annual number of new precancerous lesions and genital warts cases attributable to all HPV types and to 9-valent HPV vaccine types in Europe

The methodology used to estimate the annual number of new precancerous lesions of the cervix, vulva, vagina, and anus, as well as genital warts was comprehensively described in our previous work [5]. In the present paper we updated this estimate, as well as the fraction attributable to all HPV types and 9-valent HPV vaccine types for the 2015 population [11].

## Results

### Estimated annual number of new cancers attributable to all HPV types and to 9-valent HPV vaccine types in Europe (Table 2, Additional file 1)

**Table 2:** Estimated mean annual number of new HPV-attributable cancer cases in women and men in Europe

Cancer site	New cancers N, (95% bound)	New cancers attributable to all HPV types N, (95% bound)^a^	New cancers attributable to 9-valent HPV vaccine types (6/11/16/18/31/33/45/52/58) N, (95% bound)^a^
Cervical cancer	34,939 (32,863 – 37,032)	34,939 (32,863 – 37,032)	31,130 (28,800 – 33,495)
Vulvar cancer	9,776 (8,727 – 10,841)	1,554 (1,135 – 2,044)	1,466 (1,008 – 1,994)
Vaginal cancer	2,224 (1,723 – 2,744)	1,562 (1,058 – 2,134)	1,360 (827 – 1,980)
Anal cancer (F)	4,663 (3,968 – 5,375)	4,062 (3,203 – 4,940)	3,834 (2,851 – 4,818)
Head and neck cancers (F)	18,052 (13,977 – 22,183)	1,396 (728 – 2,592)	1,301 (586 – 2,574)
Total (women)	69,654 (61,754 – 77,609)	43,512 (39,256 – 48,387)	39,091 (34,320 – 44,513)
Anal cancer (M):	2,801 (2,260 – 3,359)	2,440 (1,822 – 3,088)	2,303 (1,621 – 3,012)
Penile cancer	4,231 (3,543 – 4,937)	1,227 (845 – 1,697)	1,113 (707 – 1,619)
Head and neck cancers (M)	81,989 (73,563 – 90,469)	5,834 (3,729 – 9,735)	5,485 (2,923 – 9,680)
Total (men)	89,021 (79,558 – 98,543)	9,501 (6,502 – 14,376)	8,901 (5,348 – 14,168)
Total (both sexes)	158,675 (141,617 – 175,815)	53,013 (45,886 – 62,589)	47,992 (39,785 – 58,511)

*HPV* human papillomavirus, *N* number, *CI* confidence interval, *F* female, *M* male

^a^Except for cervical cancer, prevalence is based on oncogenically-active HPV infections only (i.e., HPV DNA detection plus either E6^a^I mRNA expression or p16 overexpression)

#### Cervical cancer

The estimated annual number of new cervical cancers in 2015 was 34,939 (95% bound: 32,863-37,032) in the 31selected European countries combined. Applying our overall HPV attributable fraction of 100%, all of these cases are believed to be HPV-related. The fraction of cases attributable to 9-valent HPV vaccine types was 89.1% (95% CI: 87.7-90.4). Accordingly, a total of 31,130 (95% bound: 28,800-33,495) cases were estimated to be attributable to these types.

#### Vulvar cancer

The estimated annual number of new vulvar cancers was 9,776 (95% bound: 8,727-10,841). Given an overall HPV attributable fraction of 15.9% (95% CI: 13.5-18.4) [14], 1,554 (95% bound: 1,135-2,044) cases were estimated to be attributable to all HPV types. In our reference publication, the fraction of these cases attributable to 9-valent HPV vaccine types was estimated at 94.3% (95% CI: 89.1-97.5) [14]. Thus 1,466 (95% bound: 1,008-1,994) cases were estimated to be attributable to these types.

#### Vaginal cancer

The estimated annual number of new vaginal cancers was 2,224 (95% bound: 1,723-2,744). Of these cases, 1,562 (95% bound: 1,058-2,134) were estimated to be attributable to all HPV types, assuming an overall HPV attributable fraction of 70.2% (95% CI: 62.2-77.4) [16]. The fraction of these cases attributable to 9-valent HPV vaccine types in our reference publication was 87.1% (95% CI: 78.8-92.6). Applying this value, 1,360 (95% bound: 827–1,980) cases were estimated to be attributable to these types.

#### Anal cancer

The estimated annual number of new anal cancers was 4,663 (95% bound: 3,968-5,375) among women and 2,801 (95% bound: 2,260-3,359) among men in Europe. Given an overall HPV attributable fraction of 87.1% (95% CI: 81.0-91.8) [15], 4,062 cases (95% bound: 3,203-4,940) in women and 2,440 cases (95% bound: 1,822-3,088) in men were estimated to be attributable to all HPV types. The fraction of these cases attributable to 9-valent HPV vaccine types in our reference publication was 94.4% (95% CI: 89.2-97.5). After applying this value, 3,834 (95% bound: 2,851-4,818) cases in women and 2,303 (95% bound: 1,621-3,012) cases in men were estimated to be attributable to these types.

#### Penile cancer

The estimated annual number of new penile cancers was 4,231 (95% bound: 3,543-4,937). Assuming an overall HPV attributable fraction of 29.0% (95% CI: 24.7-33.7) [7], 1,227 (95% bound: 845–1,697) cases were estimated to be attributable to all HPV types. Of these cases, the fraction attributable to 9-valent HPV vaccine types in our reference publication was 90.7% (95% CI: 84.1-95.3); thus 1,113 (95% bound: 707–1,619) cases were estimated to be attributable to these types*.*

#### Head and neck cancers

The estimated annual number of new head and neck cancers was 18,052 (95% bound: 13,977-22,183) among women and 81,989 (95% bound: 73,563-90,469) among men. Of these, 1,396 (95% bound: 728–2,592) cancers in women and 5,834 (95% bound: 3,729-9,735) cancers in men were estimated to be attributable to all HPV types, given an overall HPV attributable fraction of 3.7% (95% CI: 2.4-5.6) in oral cavity cancers, 10.8% (95% CI: 3.0-25.4) in nasopharyngeal cancers, 19.9% (95% CI: 17.2-22.8) in oropharyngeal cancers, 2.4% (95% CI: 0.3-8.4) in hypopharyngeal cancers, 25.0% (95% CI: 10.7-44.9) in pharyngeal cancers, and 2.4% (95% CI: 1.2-4.1) in laryngeal cancers. The fraction attributable to 9-valent HPV vaccine types in our reference publication was 90.9% (95% CI: 70.8-98.9), 75.0% (95% CI: 19.4-99.4), 97.5% (95% CI: 93.7-99.3), 100% (95% CI: 15.8-100), 85.7% (95% CI:42.1-99.6) and 91.7% (95% CI: 61.5-99.8) for oral cavity cancers, nasopharyngeal cancers, oropharyngeal cancers, hypopharyngeal cancers, pharyngeal cancers, and laryngeal cancers, respectively. After applying these values, a total of 1,301 (95% bound: 586–2,574) head and neck cancers cases in women and 5,485 (95% bound: 2,923-9,680) in men were estimated to be attributable to 9-valent HPV vaccine types. The most frequent HPV type in head and neck cancers is HPV16, which is most commonly present as a single-type infection, but a small proportion of these cancers contain HPV18, or, even less frequently, HPV31 or 33.

### Estimated annual number of new precancerous lesions attributable to all HPV types and 9-valent HPV vaccine types in Europe

#### Cervical intraepithelial neoplasia grade 2 or worse

Based on the age-specific incidence rates, the estimated annual number of new cervical intraepithelial neoplasia grade 2 or worse (CIN2+) cases in women in Europe ranged between 263,227 and 503,010. Of these cases, 82.3% were estimated to be attributable to 9-valent HPV vaccine types. After applying these values, 216,636 to 413,977 new CIN2+ cases were estimated to be attributable to these types in 2015.

#### Vulvar intraepithelial neoplasia grades 2 and 3

Based on the age-specific incidence data, the estimated annual number of new vulvar intraepithelial neoplasia grades 2 and 3 (VIN2/3) cases was between 13,997 and 27,773. Of these cases, 86.9% (95% CI: 82.6-90.4) were estimated to be attributable to all HPV types, with 9-valent HPV vaccine types accounting for 94.4% (95% CI: 91.0-96.9) of them. Based on these estimates, 12,164 to 24,135 of the VIN2/3 cases were estimated to be attributable to all HPV types and 11,482 to 22,783 to 9-valent HPV vaccine types.

#### Vaginal intraepithelial neoplasia grades 2 and 3

Based on age-specific incidence data, the estimated annual number of new vaginal intraepithelial neoplasia grades 2 and 3 (VaIN2/3) cases in women in Europe ranged between 2,596 and 4,751. Of these cases, 95.8% (95% CI: 91.8-98.2) are expected to be attributable to all HPV types, with 9-valent HPV vaccine types accounting for 77.6% (95% CI: 70.6-83.3) of them. Based on these estimates, 2,487 to 4,551 cases were expected to be attributable to all HPV types, and 1,930-3,532 cases to 9-valent HPV vaccine types.

#### Anal intraepithelial neoplasia grades 2 and 3

Based on the age-standardized rate of 0.58 and 0.43 per 100,000 person-years for anal intraepithelial neoplasia grades 2 and 3 (AIN2/3) in women and men, respectively [17], 1,549 new AIN2/3 cases were estimated to occur each year in women and 1,097 cases in men. Of these cases, 95.3% (95% CI: 84.2-99.4%) are believed to be attributable to all HPV types [15]. Applying these values resulted in 1,477 and 1,045 cases attributable to all HPV types in women and men, respectively, of which 81.5% (95% CI: 66.4-91.9) were attributable to 9-valent HPV vaccine types, corresponding to 1,203 cases in women and 852 cases in men (Table 3).

**Table 3:** Estimated annual number of new precancerous lesions in women and men in Europe

Precancerous lesion	New precancerous lesions (range)	New precancerous lesions attributable to all HPV types (range)	New precancerous lesions attributable to 9-valent HPV vaccine types (6/11/16/18/31/33/45/52/58) (range)
CIN 2+	263,227 – 503,010	263,227 – 503,010	216,636 – 413,977
VIN 2/3	13,997 – 27,773	12,164 – 24,135	11,482 – 22,783
VaIN 2/3	2,596 – 4,751	2,487 – 4,551	1,930 – 3,532
AIN 2/3 (F)	1,549	1,477	1,203
AIN 2/3 (M)	1,097	1,045	852
Total (both sexes)	282,466 - 538,180	280,399 - 534,218	232,103 - 442,347

*HPV* human papillomavirus, *CIN2+* cervical intraepithelial neoplasia grade 2 or worse, *VIN2/3* vulvar intraepithelial neoplasia grades 2 and 3, *VaIN2/3* vaginal intraepithelial neoplasia grades 2 and 3, *AIN2/3* anal intraepithelial neoplasia grades 2 and 3, CIN 2+ includes CIN2/3 and AIS, *N* number, *F* female, *M* male

### Estimated annual number of new genital warts cases attributable to HPV in Europe

The estimated annual number of new genital warts cases ranged between 379,330 and 510,492 in women, and between 376,608 and 427,720 in men. Assuming that the low-risk 9-valent HPV vaccine types account for 90% of all genital warts cases [18], between 341,397 and 459,443 of these cases in women and between 338,947 and 384,948 of these cases in men were estimated to be attributable to these types (Table 4).

**Table 4:** Estimated annual number of new genital warts cases in women and men in Europe

	N of new annual cases (range)	N of new annual cases attributable to HPV6/11 (range)
Women	379,330 – 510,492	341,397 – 459,443
Men	376,608 – 427,720	338,947 – 384,948
Total (both sexes)	755,937 – 938,212	680,344 – 844,391

*HPV* human papillomavirus, *N* number

## Discussion

**Fig. 1:**
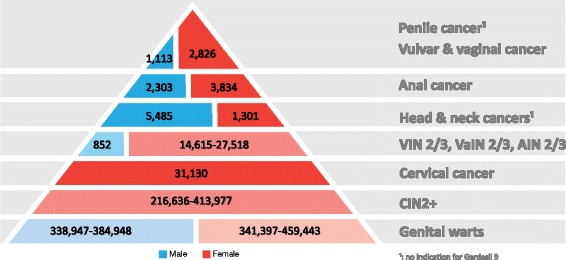
Overall burden of diseases attributable to the 9-valent HPV vaccine types in men and women in Europe. CIN2+: cervical intraepithelial neoplasia grade 2 or worse; VIN2/3: vulvar intraepithelial neoplasia grades 2 and 3; VaIN2/3: vaginal intraepithelial neoplasia grades 2 and 3; AIN2/3: anal intraepithelial neoplasia grades 2 and 3

To our knowledge, this is the first estimation of the annual number of new cancers, precancerous lesions, and genital warts attributable to 9-valent HPV vaccine types in women and men in Europe (EMA territory plus Switzerland), reflecting the overall burden of disease, including penile and head and neck cancers. Our estimates demonstrate the high disease burden associated with 9-valent HPV vaccine types (HPV6, 11, 16, 18, 31, 33, 45, 52, and 58) (Fig. 1). Among the 53,013 (95% bound: 45,886 to 62,589) new cancers attributable to all HPV types occurring yearly in Europe, 47,992 (95% bound: 39,785-58,511) are expected to be attributable to 9-valent HPV vaccine types, i.e., about 90% (see Table 2). About 81% of these cases (39,091; 95% bound: 34,320 to 44,513) occur in women and 19% (8,901; 95% bound: 5,348 to 14,168) in men. Cervical cancer represents the highest burden (31,130 cases), followed by head and neck cancer (6,786 cases), anal cancer (6,137 cases), vulvar cancer (1,466 cases), vaginal cancer (1,360 cases), and penile cancer (1,113 cases). HPV-attributable head and neck cancers constitute a heavy burden in Europe, particularly in men (1,396 cases in women, 5,834 cases in men). Overall 75–100% of these are associated with 9-valent HPV vaccine types, with HPV16 being the main contributor (50–92% attributable to this type specifically, depending on subsite).

In a previous work we estimated the incremental burden attributable to the five new vaccine types [5]; the relative increase in the number of new cancers attributable to HPV16/18/31/33/45/52/58 compared to HPV16/18 was 19%. In the present work we estimated the burden attributable to all nine HPV types and based our estimates for cancers others than cervical cancer on oncogenically-active fractions only. Also, this is the first estimate of the burden of penile and head and neck cancers. However, the additional benefit of the 9-valent vaccine compared to the quadrivalent or bivalent vaccine would not have essentially changed the earlier estimate of 19%, as head and neck cancers are mainly driven by HPV16.

In addition to cancers, 232,103 to 442,347 new precancerous lesions (CIN2+, VIN2/3, VaIN2/3, and AIN2/3) and 680,344 to 844,391 new genital warts cases (341,397 to 459,443 in women; 338,947 to 384,948 in men) per year are expected to be attributable to 9-valent HPV vaccine types. Precancerous lesions of the penis are known to be largely HPV-related: 89.1% of penile high-grade squamous intraepithelial lesions were estimated to be HPV-related in Europe, with 92% of them attributable to 9-valent HPV vaccine types [7]. However, it was not possible to find a robust data source to evaluate the incidence of penile intraepithelial neoplasia in Europe; therefore the burden associated to this disease could not be evaluated. Similarly, the burden of precancerous lesions of the head and neck could not be estimated, as no screening exists. The burden of RRP, a rare but dreadful condition that is almost exclusively attributable to HPV6 and 11, is also missing, as available incidence data are too scarce to be extrapolated to large populations [19, 20].

### Strengths and limitations

All data used to evaluate the burden of HPV-attributable cancers, precancerous lesions, and genital warts were collected during the most recent period before HPV vaccine introduction in Europe. Thus these data reflect the theoretical burden of these diseases when cervical cancer screening was the only method for disease prevention; before any impact of vaccination would be evident.

There are regional differences in the incidence of cervical cancer that are due predominantly to the combination of HPV prevalence (that depend on the cultural environment and related behavioral pattern) and the presence and effectiveness of population-based screening programs. Within Europe, the highest rates are observed in Eastern Europe, followed by Northern Europe, Southern Europe and Western Europe, where incidence is lowest. Worldwide highest incidence rates are observed in countries with low ranking of the Human Development Index and lowest rates are measured in Western Asia, Australia and the United States [21]*.*

A short-term prediction method was used to estimate the number of new cancer cases in 2015 from the most recent data collected from 2003 to 2007. These estimates presuppose that the incidence rates of the cancers under study remained stable over time. In the case of increasing incidence, as observed for anal cancers and HPV-related head and neck cancers over the last few decades [17, 22–28], this could lead to a slight underestimation of the expected number of cases. The opposite would be true in the case of decreasing incidence.

As mentioned above, the CI5 database contains national cancer incidence rates for 19 European countries. Eight of the countries included in this report had only regional incidence rates available, which were extrapolated to the entire country. Although we assessed the geographical coverage and distribution of these regional registries, other factors could vary and influence regional incidence rates. For the remaining four countries no robust regional or national data were available. We thus extrapolated the mean incidence data from surrounding cancer registries to these countries, but we had no means to check the robustness of this method. Therefore the results for those countries should be interpreted with particular caution.

Our calculations of HPV-attributable cancers other than cervical cancer were based on data provided by a large European multicenter study [6, 7, 13–16], which contains the most relevant cancer site-specific data published to-date and that provided estimates adjusted for multiple infections, in order not to overestimate the weight of individual HPV types.

Moreover, for cancers other than cervical cancer, the HPV attributable fractions were based on oncogenically-active HPV infections only (i.e., detection of HPV DNA and either mRNA and/or p16 positivity, which are markers of biological activity). Indeed, non-cervical cancers may occur for reasons other than HPV infection; the mere presence of HPV DNA is insufficient to prove causation, as the infection may be transient and not related to the carcinogenic process. This may be particularly true for head and neck cancers, for which tobacco and alcohol are known to be major risk factors. These additional criteria were implemented mainly to avoid overestimating the HPV attributable fraction in non-cervical cancers. Still, we cannot completely rule out the possibility that mRNA or protein was degraded in the paraffin-embedded samples in our reference publication, which could trigger false negativity for biological HPV activity. To evaluate this, we looked at the proportion of oncogenically-active HPV-positive samples with those that were HPV DNA-positive only, and compared it with the proportion observed in a recent meta-analysis on the same topic for head and neck cancers [29]. These proportions were consistent for oropharyngeal cancers (~87% of oncogenically-active HPV-positive cancers in the meta-analysis vs 91% in our reference study). However, this proportion was more heterogeneous for other head and neck cancers: 86% of HPV-related laryngeal cancers were considered to be attributable to HPV based on p16 detection, but this number decreased to 39% when based on mRNA in the meta-analysis (vs 61% in the study we used as reference); 28% of HPV-positive oral cavity cancers were considered to be attributable to HPV based on p16 detection, but 67% when based on mRNA in the meta-analysis (vs 59% in the reference study). It should also be noted that overall HPV DNA prevalence by subsite in the meta-analysis was higher (41.4% for oropharyngeal cancers, 20.9% for laryngeal cancers, and 17.5 for oral cavity cancers in Europe) than that observed in our reference publication (22.3% for oropharyngeal cancers, 4.8% for laryngeal cancers, and 7.8% for oral cavity cancers). Our estimated annual number of new head and neck cancers attributable to HPV should thus be considered a conservative estimate.

Some regional differences were observed in the prevalence of HPV in head and neck cancers in Europe. For example, the oncogenically-active HPV attributable fraction in oropharyngeal cancer ranged from 9.4% in Southern Europe to 50% in Northern Europe [6]. However, for the purpose of our study it was not possible to apply country-specific data, as the available data did not cover all European countries and small sample sizes would not have provided robust results.

Sex-specific data for HPV prevalence in anal cancer and head and neck cancers were available, but were not used. No sex-specific differences in HPV prevalence were seen in anal cancer (oncogenically-active HPV prevalence was 88.2 (95% CI: 76.1-95.6) in men vs 87.2 (95% CI: 79.4-92.8) in women). Even if some differences in HPV attributable fractions by sex were high in some head and neck subsites, particularly in oropharyngeal cancers (16.9% (95% CI: 14.1-20.0) in men vs 40.2% (95% CI: 31.4-49.4) in women), this was not confirmed by a recent literature review on the topic [30]. According to the analysis of Combes et al., there are regional differences in the sex-specific HPV prevalence of oropharyngeal cancers worldwide and within Europe (male:female HPV prevalence ratio <1 in France, Germany, Italy, and the Czech Republic; male:female ratio ≥1 in the Netherlands, Norway, the United Kingdom, and Sweden). Still, when considering Europe as a whole, and based on 27 studies conducted in 9 countries of the European Union with less than 200 subjects each, the male:female ratio for HPV prevalence in oropharyngeal cancers was 1.0 (0.9-1.1), and the estimated HPV prevalence in oropharyngeal cancers was 40.3 in males and 41.2 in females. In addition, that paper suggested that smoking and heavy drinking may either enhance the carcinogenic effect of HPV or hamper the accurate attribution of oropharyngeal cancers to HPV in men who have both the infection and exhibit the two lifestyle risk factors related to this disease. The sex-specific differences in HPV prevalence in oropharyngeal cancer are mainly a consequence of the vast international variation in male smoking habits. According to these results, and as the difference in the male:female ratio observed in our reference publication may be due to chance (regional representativity of participating countries), we finally decided to use a single HPV attributable fraction for oropharyngeal cancer for males and females.

Moreover, a declining incidence of HPV-negative oropharyngeal squamous cell carcinoma is currently being observed in the United States that parallels with declines in smoking. In contrast, increasing incidence of HPV-positive OPSCCs perhaps arises from increased oral sex and oral HPV exposure over calendar time. Indeed, prevalence of genital herpes simplex virus 1 (HSV1), HSV2, and genital warts have increased among recent birth cohorts in the United States, accepted surrogates for oral sex, risky sexual behavior, and HPV exposure, respectively. The predominant rise in OPSCC incidence among the young is also consistent with changing HPV exposure among recent birth cohorts. However, the reasons for pronounced increases among men remain unexplained [31]. Therefore it is critical to understand if there is an underlying epidemic of HPV-positive head and neck cancers related to changes in sexual habits, because this could change all our forecasts of the HPV- related epidemiology of head and neck cancer.

A further limitation is represented by the fact that only one-digit ICD codes are available in the CI5 database, meaning that some of the head and neck cancer subsites could not be correctly assigned. For example C.5.1 (soft palate), C.5.2 (uvula) were classified as oral cancers and C.14.2 (Waldeyers ring) as pharyngeal cancers, but anatomically they all belong to the oropharynx.

The method used to calculate the estimated annual number of new precancerous lesions also has some limitations that were described in our previous estimation of HPV-related burden of disease [5].

To our knowledge, the data that we used for our estimation of cancers, precancerous lesions, and genital warts are the most robust data available in Europe to-date. However, the results of this evaluation have to be considered with caution, as several extrapolations and assumptions were used. Future studies are necessary; mainly to further evaluate the HPV attributable fraction of head and neck cancers and the real burden of precancerous lesions of the vulva, vagina, and anus, for which no systematic screening is performed. Additionally, incidence data are completely lacking for precancerous lesions of the penis and in the head and neck area and are very scarce for RRP.

## Conclusions

The burden of cancers attributable to 9-valent HPV vaccine types in Europe is substantial, both in women and men. Overall, 53,013 (95% bound: 48,160-67,171) HPV-attributable cancers were estimated to occur every year in Europe, of which more than 90%, (47,992 (95% bound: 39,785-58,511)) were estimated to be attributable to 9-valent HPV vaccine HPV types. When considering head and neck cancers in addition to anogenital cancers, about 19% of all HPV-attributable cancers are expected to occur in men, and most of these cancers are attributable to 9-valent HPV vaccine types. In addition to cancers, 232,103 to 442,347 new cases of precancerous lesions (CIN2/3, adenocarcinoma *in situ*, VIN2/3, VaIN2/3, and AIN2/3) and 680,344 to 844,391 new genital warts cases (341,397 to 459,443 in women, 338,947 to 384,948 in men) are expected to be attributable to the 9-valent HPV vaccine types each year. This data demonstrates the important preventive potential of the new 9-valent HPV vaccine in Europe.

## Additional file

Additional file 1: Annex 1. Estimated mean annual number of new HPV-related cancer cases in women and men per European country. (DOCX 57 kb)

## Abbreviations

AIN2/3: Anal intraepithelial neoplasia grades 2 and 3
CI: Confidence interval
CI5: Cancer incidence in five continents
CIN2+: Cervical intraepithelial neoplasia grade 2 or worse
EMA: European Medicines Agency
HPV: Human papillomavirus
IARC: International Agency for Research on Cancer
ICD-10: International classification of diseases 10^th^ revision
RRP: Recurrent respiratory papillomatosis
VaIN2/3: Vaginal intraepithelial neoplasia grades 2 and 3
VIN2/3: Vulvar intraepithelial neoplasia grades 2 and 3

## References

[1] Joura EA, Giuliano AR, Iversen OE, Bouchard C, Mao C, Mehlsen J (2015). A 9-valent HPV vaccine against infection and intraepithelial neoplasia in women. N Engl J Med.

[2] National Institutes of Health, National Cancer Institute. Gardasil 9 Vaccine Protects against Additional HPV Types. https://www.cancer.gov/types/cervical/research/gardasil9-prevents-more-HPV-types. Accessed 30 Nov 2016

[3] Vichnin M, Bonanni P, Klein NP, Garland SM, Block SL, Kjaer SK (2015). An overview of quadrivalent human papillomavirus vaccine safety: 2006 to 2015. Pediatr Infect Dis J.

[4] European Medicines Agency. Annexe I. Summary of Product Characteristics. http://www.ema.europa.eu/docs/en_GB/document_library/EPAR_-_Product_Information/human/003852/WC500189111.pdf. Accessed 30 Nov 2016.

[5] Hartwig S, Baldauf JJ, Dominiak-Felden G, Simondon F, Alemany L, de Sanjose S (2015). Estimation of the epidemiological burden of HPV-related anogenital cancers, precancerous lesions, and genital warts in women and men in Europe: potential additional benefit of a nine-valent second generation HPV vaccine compared to first generation HPV vaccines. Papillomavirus Res.

[6] Castellsague X, Alemany L, Quer M, Halec G, Quiros B, Tous S (2016). HPV involvement in head and neck cancers: comprehensive assessment of biomarkers in 3680 patients. J Natl Cancer Inst.

[7] Alemany L, Cubilla A, Halec G, Kasamatsu E, Quiros B, Masferrer E (2016). Role of human papillomavirus in penile carcinomas worldwide. Eur Urol.

[8] International Agency for Research on Cancer. www.iarc.fr. Accessed 30 Nov 2016.

[9] IARC. Globocan 2012: Estimated cancer incidence, mortality and prevalence worldwide in 2012. Data sources and methods. http://globocan.iarc.fr/Pages/DataSource_and_methods.aspx. Accessed 30 Nov 2016.

[10] Forman D, Bray F, Brewster DH, Gombe Mbalawa C, Kohler B, Piñeros M (2013). Cancer incidence in five continents.

[11] European Commission: Eurostat homepage. http://ec.europa.eu/eurostat/data/database. Accessed 30 Nov.

[12] Walboomers JM, Jacobs MV, Manos MM, Bosch FX, Kummer JA, Shah KV (1999). Human papillomavirus is a necessary cause of invasive cervical cancer worldwide. J Pathol.

[13] de Sanjosé S, Quint WG, Alemany L, Geraets DT, Klaustermeier JE, Lloveras B (2010). Human papillomavirus genotype attribution in invasive cervical cancer: a retrospective cross-sectional worldwide study. Lancet Oncol.

[14] de Sanjosé S, Alemany L, Ordi J, Tous S, Alejo M, Bigby SM (2013). Worldwide human papillomavirus genotype attribution in over 2000 cases of intraepithelial and invasive lesions of the vulva. Eur J Cancer.

[15] Alemany L, Saunier M, Alvarado-Cabrero I, Quiros B, Salmeron J, Shin HR (2015). Human papillomavirus DNA prevalence and type distribution in anal carcinomas worldwide. Int J Cancer.

[16] Alemany L, Saunier M, Tinoco L, Quiros B, Alvarado-Cabrero I, Alejo M (2014). Large contribution of human papillomavirus in vaginal neoplastic lesions: a worldwide study in 597 samples. Eur J Cancer.

[17] Nielsen A, Munk C, Kjaer SK (2012). Trends in incidence of anal cancer and high-grade anal intraepithelial neoplasia in Denmark, 1978–2008. Int J Cancer.

[18] European Centers for Disease Prevention and Control (2012). Introduction of HPV vaccines in EU countries - an update.

[19] Lindeberg H, Elbrond O (1990). Laryngeal papillomas: the epidemiology in a Danish subpopulation 1965–1984. Clin Otolaryngol Allied Sci.

[20] Silverberg MJ, Thorsen P, Lindeberg H, Grant LA, Shah KV (2003). Condyloma in pregnancy is strongly predictive of juvenile-onset recurrent respiratory papillomatosis. Obstet Gynecol.

[21] Forman D, de MC, Lacey CJ, Soerjomataram I, Lortet-Tieulent J, Bruni L (2012). Global burden of human papillomavirus and related diseases. Vaccine.

[22] Habbous S, Chu KP, Qiu X, La DA, Harland LT, Fadhel E (2013). The changing incidence of human papillomavirus-associated oropharyngeal cancer using multiple imputation from 2000 to 2010 at a Comprehensive Cancer Centre. Cancer Epidemiol.

[23] McCarthy CE, Field JK, Rajlawat BP, Field AE, Marcus MW (2015). Trends and regional variation in the incidence of head and neck cancers in England: 2002 to 2011. Int J Oncol.

[24] Annertz K, Anderson H, Palmer K, Wennerberg J (2012). The increase in incidence of cancer of the tongue in the Nordic countries continues into the twenty-first century. Acta Otolaryngol.

[25] Brewster DH, Bhatti LA (2006). Increasing incidence of squamous cell carcinoma of the anus in Scotland, 1975–2002. Br J Cancer.

[26] Robinson D, Coupland V, Moller H (2009). An analysis of temporal and generational trends in the incidence of anal and other HPV-related cancers in Southeast England. Br J Cancer.

[27] Goldman S, Glimelius B, Nilsson B, Pahlman L (1989). Incidence of anal epidermoid carcinoma in Sweden 1970–1984. Acta Chir Scand.

[28] Van Lieshout A, Pronk A (2010). [Increasing incidence of anal cancer in the Netherlands]. Ned Tijdschr Geneeskd.

[29] Ndiaye C, Mena M, Alemany L, Arbyn M, Castellsague X, Laporte L (2014). HPV DNA, E6/E7 mRNA, and p16INK4a detection in head and neck cancers: a systematic review and meta-analysis. Lancet Oncol.

[30] Combes JD, Chen AA, Franceschi S (2014). Prevalence of human papillomavirus in cancer of the oropharynx by gender. Cancer Epidemiol Biomarkers Prev.

[31] Chaturvedi AK, Engels EA, Pfeiffer RM, Hernandez BY, Xiao W, Kim E (2011). Human papillomavirus and rising oropharyngeal cancer incidence in the United States. J Clin Oncol.

