# Supplementary material for: Estimation of the overall burden of cancers, precancerous lesions, and genital warts attributable to 9-valent HPV vaccine types in women and men in Europe
Source: Infect Agent Cancer. 2017 Apr 11;12:19. doi: 10.1186/s13027-017-0129-6 (PMC5387299; doi:10.1186/s13027-017-0129-6)
Supplement: Additional file 1: Annex 1. — Estimated mean annual number of new HPV-related cancer cases in women and men per European country. (DOCX 57 kb) [file 13027_2017_129_MOESM1_ESM.docx]

**Annex 1 - Estimated mean annual number of new HPV-related cancer cases in women and men per European country**

**A Cervical cancer**

| **Country** | **N of new cancers irrespective of HPV status (95% CI)** | **N of new cancers attributable to HPV (95% CI)^1^** | **N of HPV6/11/16/18/31/33/45/52/58+ cancers (95% CI)^2^** |
| --- | --- | --- | --- |
| Austria | 635 (586 - 684) | 635 (586 - 684) | 566 (519 - 613) |
| Belgium | 699 (647 - 751) | 699 (647 - 751) | 623 (574 - 672) |
| Bulgaria | 1089 (1024 - 1154) | 1089 (1024 - 1154) | 970 (909 - 1031) |
| Croatia | 344 (308 - 380) | 344 (308 - 380) | 306 (272 - 340) |
| Cyprus | 28 (18 - 38) | 28 (18 - 38) | 25 (15 - 35) |
| Czech Republic | 1106 (1041 - 1171) | 1106 (1041 - 1171) | 986 (924 - 1048) |
| Denmark | 402 (363 - 441) | 402 (363 - 441) | 358 (321 - 395) |
| Estonia | 164 (139 - 189) | 164 (139 - 189) | 146 (122 - 170) |
| Finland | 166 (141 - 191) | 166 (141 - 191) | 148 (124 - 172) |
| France | 3454 (3339 - 3569) | 3454 (3339 - 3569) | 3078 (2969 - 3187) |
| Germany | 5255 (5113 - 5397) | 5255 (5113 - 5397) | 4682 (4548 - 4816) |
| Greece | 1493 (1417 - 1569) | 1493 (1417 - 1569) | 1330 (1259 - 1401) |
| Hungary | 987 (925 - 1049) | 987 (925 - 1049) | 879 (821 - 937) |
| Iceland | 15 (8 - 25) | 15 (8 - 25) | 14 (8 - 23) |
| Ireland | 284 (251 - 317) | 284 (251 - 317) | 253 (222 - 284) |
| Italy | 2708 (2606 - 2810) | 2708 (2606 - 2810) | 2413 (2317 - 2509) |
| Latvia | 213 (184 - 242) | 213 (184 - 242) | 189 (162 - 216) |
| Lithuania | 484 (441 - 527) | 484 (441 - 527) | 431 (390 - 472) |
| Luxembourg | 32 (21 - 43) | 32 (21 - 43) | 28 (18 - 38) |
| Malta | 12 (6 - 21) | 12 (6 - 21) | 11 (5 - 20) |
| Norway | 321 (286 - 356) | 321 (286 - 356) | 286 (253 - 319) |
| Poland | 3697 (3578 - 3816) | 3697 (3578 - 3816) | 3294 (3182 - 3406) |
| Portugal | 1013 (951 - 1075) | 1013 (951 - 1075) | 903 (844 - 962) |
| Romania | 2692 (2590 - 2794) | 2692 (2590 - 2794) | 2398 (2302 - 2494) |
| Slovakia | 632 (583 - 681) | 632 (583 - 681) | 563 (516 - 610) |
| Slovenia | 180 (154 - 206) | 180 (154 - 206) | 161 (136 - 186) |
| Spain | 2336 (2241 - 2431) | 2336 (2241 - 2431) | 2082 (1993 - 2171) |
| Sweden | 474 (431 - 517) | 474 (431 - 517) | 423 (383 - 463) |
| Switzerland | 268 (236 - 300) | 268 (236 - 300) | 238 (208 - 268) |
| The Netherlands | 707 (655 - 759) | 707 (655 - 759) | 630 (581 - 679) |
| United Kingdom | 3050 (2942 - 3158) | 3050 (2942 - 3158) | 2718 (2616 - 2820) |

^1^ HPV prevalence: 100%, ^2^ HPV 6/11/16/18/31/33/45/52/58 attributable fraction among HPV+ cases: 89.1% (87.7–90.4) (ref: de Sanjosé et al, Lancet Oncol, 2010)

HPV: human papillomavirus; CI: confidence interval

**B Vulvar cancer**

| **Country** | **N of new cancers irrespective of HPV status (95% CI)** | **N of new cancers attributable to HPV (mRNA and/or p16 positive), (95% CI)^3^** | **N of new cancers attributable to HPV 6/11/16/18/31/33/45/52/58 (mRNA and/or p16 positive), (95% CI)^4^** |
| --- | --- | --- | --- |
| Austria | 139 (116 - 162) | 22 (13 - 31) | 21 (12 - 30) |
| Belgium | 201 (173 - 229) | 32 (21 - 43) | 30 (19 - 41) |
| Bulgaria | 130 (108 - 152) | 21 (12 - 30) | 19 (11 - 30) |
| Croatia | 77 (60 - 94) | 12 (6 - 21) | 11 (5 - 20) |
| Cyprus | 10 (5 - 18) | 2 (0 - 7) | 1 (0 - 6) |
| Czech Republic | 232 (202 - 262) | 37 (25 - 49) | 35 (23 - 47) |
| Denmark | 104 (84 - 124) | 17 (10 - 27) | 16 (9 - 26) |
| Estonia | 30 (19 - 41) | 5 (2 - 12) | 5 (2 - 12) |
| Finland | 86 (68 - 104) | 14 (8 - 23) | 13 (7 - 22) |
| France | 777 (722 - 832) | 124 (102 - 146) | 117 (96 - 138) |
| Germany | 2510 (2412 - 2608) | 399 (360 - 438) | 376 (338 - 414) |
| Greece | 192 (165 - 219) | 31 (20 - 42) | 29 (18 - 40) |
| Hungary | 199 (171 - 227) | 32 (21 - 43) | 30 (19 - 41) |
| Iceland | 3 (1 - 9) | 0 (0 - 4) | 0 (0 - 4) |
| Ireland | 54 (40 - 68) | 9 (4 - 17) | 8 (3 - 16) |
| Italy | 1175 (1108 - 1242) | 187 (160 - 214) | 176 (150 - 202) |
| Latvia | 43 (30 - 56) | 7 (3 - 14) | 6 (2 - 13) |
| Lithuania | 60 (45 - 75) | 10 (5 - 18) | 9 (4 - 17) |
| Luxembourg | 11 (5 - 20) | 2 (0 - 7) | 2 (0 - 7) |
| Malta | 10 (5 - 18) | 2 (0 - 7) | 1 (0 - 6) |
| Norway | 100 (80 - 120) | 16 (9 - 26) | 15 (8 - 25) |
| Poland | 516 (471 - 561) | 82 (64 - 100) | 77 (60 - 94) |
| Portugal | 112 (91 - 133) | 18 (11 - 28) | 17 (10 - 27) |
| Romania | 331 (295 - 367) | 53 (39 - 67) | 50 (36 - 64) |
| Slovakia | 82 (64 - 100) | 13 (7 - 22) | 12 (6 - 21) |
| Slovenia | 42 (29 - 55) | 7 (3 - 14) | 6 (2 - 13) |
| Spain | 726 (673 - 779) | 115 (94 - 136) | 109 (89 - 129) |
| Sweden | 173 (147 - 199) | 27 (17 - 37) | 26 (16 - 36) |
| Switzer-land | 131 (109 - 153) | 21 (12 - 30) | 20 (11 - 29) |
| The Nether-lands | 328 (293 - 363) | 52 (38 - 66) | 49 (35 - 63) |
| United Kingdom | 1193 (1125 - 1261) | 190 (163 - 217) | 179 (153 - 205) |

^3^ HPV DNA and (mRNA or p16) prevalence %: 15.9% (13.5-18.4), ^4^ HPV DNA and (mRNA or p16) 6/11/18/31/33/45/52/58 attributable fraction among HPV DNA and (mRNA or p16) + cases: 94.3% (89.1-97.5) (ref: de Sanjosé et al, Eur J Cancer, 2013)

HPV: human papillomavirus; CI: confidence interval

**C Vaginal cancer**

| **Country** | **N of new cancers irrespective of HPV status (95% CI)** | **N of new cancers attributable to HPV (mRNA and/or p16 positive), (95% CI)^5^** | **N of new cancers attributable to HPV 6/11/16/18/31/33/45/52/58 (mRNA and/or p16 positive), (95% CI)^6^** |
| --- | --- | --- | --- |
| Austria | 65 (49 - 81) | 45 (32 - 58) | 39 (27 - 51) |
| Belgium | 55 (40 - 70) | 39 (27 - 51) | 34 (23 - 45) |
| Bulgaria | 29 (18 - 40) | 20 (11 - 29) | 18 (11 - 28) |
| Croatia | 17 (10 - 27) | 12 (6 - 21) | 10 (5 - 18) |
| Cyprus | 1 (0 - 6) | 1 (0 - 6) | 0 (0 - 4) |
| Czech Republic | 55 (40 - 70) | 38 (26 - 50) | 33 (22 - 44) |
| Denmark | 27 (17 - 37) | 19 (11 - 30) | 17 (10 - 27) |
| Estonia | 6 (2 - 13) | 4 (1 - 10) | 4 (1 - 10) |
| Finland | 22 (13 - 31) | 16 (9 - 26) | 14 (8 - 23) |
| France | 238 (208 - 268) | 167 (142 - 192) | 146 (122 - 170) |
| Germany | 482 (439 - 525) | 339 (303 - 375) | 295 (261 - 329) |
| Greece | 45 (32 - 58) | 31 (20 - 42) | 27 (17 - 37) |
| Hungary | 50 (36 - 64) | 35 (23 - 47) | 30 (19 - 41) |
| Iceland | 0 (0 - 4) | 0 (0 - 4) | 0 (0 - 4) |
| Ireland | 15 (8 - 25) | 10 (5 - 18) | 9 (4 - 17) |
| Italy | 248 (217 - 279) | 174 (148 - 200) | 152 (128 - 176) |
| Latvia | 9 (4 - 17) | 6 (2 - 13) | 5 (2 - 12) |
| Lithuania | 11 (5 - 20) | 8 (3 - 16) | 7 (3 - 14) |
| Luxembourg | 2 (0 - 7) | 2 (0 - 7) | 1 (0 - 6) |
| Malta | 1 (0 - 6) | 1 (0 - 6) | 1 (0 - 6) |
| Norway | 17 (10 - 27) | 12 (6 - 21) | 11 (5 - 20) |
| Poland | 118 (97 - 139) | 83 (65 - 101) | 72 (55 - 89) |
| Portugal | 75 (58 - 92) | 53 (39 - 67) | 46 (33 - 59) |
| Romania | 78 (61 - 95) | 55 (40 - 70) | 48 (34 - 62) |
| Slovakia | 22 (13 - 31) | 16 (9 - 26) | 14 (8 - 23) |
| Slovenia | 8 (3 - 16) | 6 (2 - 13) | 5 (2 - 12) |
| Spain | 124 (102 - 146) | 87 (69 - 105) | 76 (59 - 93) |
| Sweden | 44 (31 - 57) | 31 (20 - 42) | 27 (17 - 37) |
| Switzerland | 32 (21 - 43) | 23 (14 - 32) | 20 (11 - 29) |
| The Netherlands | 58 (43 - 73) | 41 (28 - 54) | 35 (23 - 47) |
| United Kingdom | 269 (237 - 301) | 189 (162 - 216) | 164 (139 - 189) |

^5^ HPV DNA and (mRNA or p16) prevalence %: 70.2% (62.2-77.4), ^6^ HPV DNA and (mRNA or p16) 6/11/18/31/33/45/52/58 attributable fraction among HPV DNA and (mRNA or p16) + cases: 87.1% (78.8-92.6) (ref: Alemany et al, Eur J Cancer, 2014)

HPV: human papillomavirus; CI: confidence interval

**D Anal cancer**

| **Country** | **Sex** | **N of new cancers irrespective of HPV status (95% CI)** | **N of new cancers attributable to HPV (mRNA and/or p16 positive), (95% CI)^7^** | **N of new cancers attributable to HPV 6/11/16/18/31/33/45/52/58 (mRNA and/or p16 positive), (95% CI)^8^** |
| --- | --- | --- | --- | --- |
| Austria | Women | 100 (80 - 120) | 87 (69 - 105) | 82 (64 - 100) |
|  | Men | 40 (28 - 52) | 35 (23 - 47) | 33 (22 - 44) |
|  | Both sexes | 139 (116 - 162) | 122 (100 - 144) | 115 (94 - 136) |
| Belgium | Women | 88 (70 - 106) | 77 (60 - 94) | 72 (55 - 89) |
|  | Men | 60 (45 - 75) | 52 (38 - 66) | 49 (35 - 63) |
|  | Both sexes | 148 (124 - 172) | 129 (107 - 151) | 121 (99 - 143) |
| Bulgaria | Women | 26 (16 - 36) | 22 (13 - 31) | 21 (12 - 30) |
|  | Men | 34 (23 - 45) | 30 (19 - 41) | 28 (18 - 38) |
|  | Both sexes | 60 (45 - 75) | 52 (38 - 66) | 49 (35 - 63) |
| Croatia | Women | 16 (9 - 26) | 14 (8 - 23) | 13 (7 - 22) |
|  | Men | 9 (4 - 17) | 8 (3 - 16) | 7 (3 - 14) |
|  | Both sexes | 25 (15 - 35) | 22 (13 - 31) | 20 (11 - 29) |
| Cyprus | Women | 3 (1 - 9) | 3 (1 - 9) | 2 (0 - 7) |
|  | Men | 2 (0 - 7) | 2 (0 - 7) | 2 (0 - 7) |
|  | Both sexes | 5 (2 - 12) | 4 (1 - 10) | 4 (1 - 10) |
| Czech Republic | Women | 78 (61 - 95) | 68 (52 - 84) | 64 (48 - 80) |
|  | Men | 46 (33 - 59) | 40 (28 - 52) | 38 (26 - 50) |
|  | Both sexes | 124 (102 - 146) | 108 (88 - 128) | 102 (82 - 122) |
| Denmark | Women | 73 (56 - 90) | 64 (48 - 80) | 60 (45 - 75) |
|  | Men | 36 (24 - 48) | 31 (20 - 42) | 29 (18 - 40) |
|  | Both sexes | 109 (89 - 129) | 95 (76 - 114) | 90 (71 - 109) |
| Estonia | Women | 10 (5 - 18) | 9 (4 - 17) | 9 (4 - 17) |
|  | Men | 4 (1 - 10) | 3 (1 - 9) | 3 (1 - 9) |
|  | Both sexes | 14 (8 - 23) | 12 (6 - 21) | 12 (6 - 21) |
| Finland | Women | 25 (15 - 35) | 22 (13 - 31) | 21 (12 - 30) |
|  | Men | 16 (9 - 26) | 14 (8 - 23) | 13 (7 - 22) |
|  | Both sexes | 41 (28 - 54) | 36 (24 - 48) | 34 (23 - 45) |
| France | Women | 824 (768 - 880) | 717 (665 - 769) | 677 (626 - 728) |
|  | Men | 322 (287 - 357) | 281 (248 - 314) | 265 (233 - 297) |
|  | Both sexes | 1146 (1080 - 1212) | 998 (936 - 1060) | 942 (882 - 1002) |
| Germany | Women | 1030 (967 - 1093) | 897 (838 - 956) | 847 (790 - 904) |
|  | Men | 589 (541 - 637) | 513 (469 - 557) | 484 (441 - 527) |
|  | Both sexes | 1619 (1540 - 1698) | 1410 (1336 - 1484) | 1331 (1259 - 1403) |
| Greece | Women | 46 (33 - 59) | 40 (28 - 52) | 38 (26 - 50) |
|  | Men | 52 (38 - 66) | 45 (32 - 58) | 42 (29 - 55) |
|  | Both sexes | 97 (78 - 116) | 85 (67 - 103) | 80 (62 - 98) |
| Hungary | Women | 59 (44 - 74) | 51 (37 - 65) | 48 (34 - 62) |
|  | Men | 31 (20 - 42) | 27 (17 - 37) | 25 (15 - 35) |
|  | Both sexes | 90 (71 - 109) | 78 (61 - 95) | 74 (57 - 91) |
| Iceland | Women | 3 (1 - 9) | 2 (0 - 7) | 2 (0 - 7) |
|  | Men | 2 (0 - 7) | 1 (0 - 6) | 1 (0 - 6) |
|  | Both sexes | 4 (1 - 10) | 4 (1 - 10) | 3 (1 - 9) |
| Ireland | Women | 23 (14 - 32) | 20 (11 - 29) | 19 (11 - 30) |
|  | Men | 16 (9 - 26) | 14 (8 - 23) | 13 (7 - 22) |
|  | Both sexes | 40 (28 - 52) | 35 (23 - 47) | 33 (22 - 44) |
| Italy | Women | 622 (573 - 671) | 542 (496 - 588) | 511 (467 - 555) |
|  | Men | 436 (395 - 477) | 380 (342 - 418) | 359 (322 - 396) |
|  | Both sexes | 1058 (994 - 1122) | 922 (862 - 982) | 870 (812 - 928) |
| Latvia | Women | 10 (5 - 18) | 9 (4 - 17) | 8 (3 - 16) |
|  | Men | 6 (2 - 13) | 5 (2 - 12) | 5 (2 - 12) |
|  | Both sexes | 16 (9 - 26) | 14 (8 - 23) | 13 (7 - 22) |
| Lithuania | Women | 11 (5 - 20) | 9 (4 - 17) | 9 (4 - 17) |
|  | Men | 8 (3 - 16) | 7 (3 - 14) | 6 (2 - 13) |
|  | Both sexes | 19 (11 - 30) | 16 (9 - 26) | 15 (8 - 25) |
| Luxem-bourg | Women | 6 (2 - 13) | 5 (2 - 12) | 5 (2 - 12) |
|  | Men | 3 (1 - 9) | 3 (1 - 9) | 3 (1 - 9) |
|  | Both sexes | 9 (4 - 17) | 8 (3 - 16) | 7 (3 - 14) |
| Malta | Women | 2 (0 - 7) | 2 (0 - 7) | 2 (0 - 7) |
|  | Men | 1 (0 - 6) | 1 (0 - 6) | 1 (0 - 6) |
|  | Both sexes | 3 (1 - 9) | 2 (0 - 7) | 2 (0 - 7) |
| Norway | Women | 51 (37 - 65) | 44 (31 - 57) | 42 (29 - 55) |
|  | Men | 23 (14 - 32) | 20 (11 - 29) | 19 (11 - 30) |
|  | Both sexes | 74 (57 - 91) | 64 (48 - 80) | 61 (46 - 76) |
| Poland | Women | 238 (208 - 268) | 208 (180 - 236) | 196 (169 - 223) |
|  | Men | 116 (95 - 137) | 101 (81 - 121) | 95 (76 - 114) |
|  | Both sexes | 354 (317 - 391) | 308 (274 - 342) | 291 (258 - 324) |
| Portugal | Women | 88 (70 - 106) | 77 (60 - 94) | 72 (55 - 89) |
|  | Men | 37 (25 - 49) | 33 (22 - 44) | 31 (20 - 42) |
|  | Both sexes | 126 (104 - 148) | 109 (89 - 129) | 103 (83 - 123) |
| Romania | Women | 76 (59 - 93) | 66 (50 - 82) | 63 (47 - 79) |
|  | Men | 77 (60 - 94) | 67 (51 - 83) | 63 (47 - 79) |
|  | Both sexes | 153 (129 - 177) | 133 (110 - 156) | 126 (104 - 148) |
| Slovakia | Women | 24 (14 - 34) | 21 (12 - 30) | 19 (11 - 30) |
|  | Men | 15 (8 - 25) | 13 (7 - 22) | 12 (6 - 21) |
|  | Both sexes | 39 (27 - 51) | 34 (23 - 45) | 32 (21 - 43) |
| Slovenia | Women | 10 (5 - 18) | 9 (4 - 17) | 8 (3 - 16) |
|  | Men | 11 (5 - 20) | 9 (4 - 17) | 9 (4 - 17) |
|  | Both sexes | 21 (12 - 30) | 18 (11 - 28) | 17 (10 - 27) |
| Spain | Women | 178 (152 - 204) | 155 (131 - 179) | 146 (122 - 170) |
|  | Men | 223 (194 - 252) | 194 (167 - 221) | 183 (156 - 210) |
|  | Both sexes | 401 (362 - 440) | 349 (312 - 386) | 330 (294 - 366) |
| Sweden | Women | 99 (79 - 119) | 87 (69 - 105) | 82 (64 - 100) |
|  | Men | 45 (32 - 58) | 39 (27 - 51) | 37 (25 - 49) |
|  | Both sexes | 145 (121 - 169) | 126 (104 - 148) | 119 (98 - 140) |
| Switzer-land | Women | 147 (123 - 171) | 128 (106 - 150) | 121 (99 - 143) |
|  | Men | 58 (43 - 73) | 50 (36 - 64) | 48 (34 - 62) |
|  | Both sexes | 205 (177 - 233) | 179 (153 - 205) | 169 (144 - 194) |
| The Nether-lands | Women | 85 (67 - 103) | 74 (57 - 91) | 70 (54 - 86) |
|  | Men | 70 (54 - 86) | 61 (46 - 76) | 58 (43 - 73) |
|  | Both sexes | 156 (132 - 180) | 136 (113 - 159) | 128 (106 - 150) |
| United Kingdom | Women | 612 (564 - 660) | 533 (488 - 578) | 503 (459 - 547) |
|  | Men | 414 (374 - 454) | 361 (324 - 398) | 341 (305 - 377) |
|  | Both sexes | 1027 (964 - 1090) | 894 (835 - 953) | 844 (787 - 901) |

^7^ HPV DNA and (mRNA or p16) prevalence %: 87.1% (81.0-91.8), ^8^ HPV DNA and (mRNA or p16) 6/11/18/31/33/45/52/58 attributable fraction among HPV DNA and (mRNA or p16) + cases: 94.4% (89.2-97.5) (ref: Alemany et al, Int J Cancer, 2014)

HPV: human papillomavirus; CI: confidence interval

**E Penile cancer**

| **Country** | **N of new cancers irrespective of HPV status (95% CI)** | **N of new cancers attributable to HPV (mRNA and/or p16 positive), (95% CI)^9^** | **N of new cancers attributable to HPV 6/11/16/18/31/33/45/52/58 (mRNA and/or p16 positive), (95% CI)^10^** |
| --- | --- | --- | --- |
| Austria | 36 (24 - 48) | 11 (5 - 20) | 10 (5 - 18) |
| Belgium | 85 (67 - 103) | 25 (15 - 35) | 22 (13 - 31) |
| Bulgaria | 48 (34 - 62) | 14 (8 - 23) | 13 (7 - 22) |
| Croatia | 24 (14 - 34) | 7 (3 - 14) | 6 (2 - 13) |
| Cyprus | 6 (2 - 13) | 2 (0 - 7) | 2 (0 - 7) |
| Czech Republic | 96 (77 - 115) | 28 (18 - 38) | 25 (15 - 35) |
| Denmark | 55 (40 - 70) | 16 (9 - 26) | 15 (8 - 25) |
| Estonia | 10 (5 - 18) | 3 (1 - 9) | 3 (1 - 9) |
| Finland | 29 (18 - 40) | 8 (3 - 16) | 8 (3 - 16) |
| France | 475 (432 - 518) | 138 (115 - 161) | 125 (103 - 147) |
| Germany | 787 (732 - 842) | 228 (198 - 258) | 207 (179 - 235) |
| Greece | 80 (62 - 98) | 23 (14 - 32) | 21 (12 - 30) |
| Hungary | 59 (44 - 74) | 17 (10 - 27) | 15 (8 - 25) |
| Iceland | 3 (1 - 9) | 1 (0 - 6) | 1 (0 - 6) |
| Ireland | 29 (18 - 40) | 8 (3 - 16) | 8 (3 - 16) |
| Italy | 492 (449 - 535) | 143 (120 - 166) | 129 (107 - 151) |
| Latvia | 16 (9 - 26) | 5 (2 - 12) | 4 (1 - 10) |
| Lithuania | 23 (14 - 32) | 7 (3 - 14) | 6 (2 - 13) |
| Luxembourg | 4 (1 - 10) | 1 (0 - 6) | 1 (0 - 6) |
| Malta | 4 (1 - 10) | 1 (0 - 6) | 1 (0 - 6) |
| Norway | 48 (34 - 62) | 14 (8 - 23) | 13 (7 - 22) |
| Poland | 227 (197 - 257) | 66 (50 - 82) | 60 (45 - 75) |
| Portugal | 76 (59 - 93) | 22 (13 - 31) | 20 (11 - 29) |
| Romania | 130 (108 - 152) | 38 (26 - 50) | 34 (23 - 45) |
| Slovakia | 35 (23 - 47) | 10 (5 - 18) | 9 (4 - 17) |
| Slovenia | 12 (6 - 21) | 3 (1 - 9) | 3 (1 - 9) |
| Spain | 504 (460 - 548) | 146 (122 - 170) | 133 (110 - 156) |
| Sweden | 95 (76 - 114) | 28 (18 - 38) | 25 (15 - 35) |
| Switzerland | 62 (47 - 77) | 18 (11 - 28) | 16 (9 - 26) |
| The Netherlands | 144 (120 - 168) | 42 (29 - 55) | 38 (26 - 50) |
| United Kingdom | 536 (491 - 581) | 155 (131 - 179) | 141 (118 - 164) |

^9^ HPV DNA and (mRNA or p16) prevalence %: 29.0% (24.7-33.7), ^10^ HPV DNA and (mRNA or p16) 6/11/18/31/33/45/52/58 attributable fraction among HPV DNA and (mRNA or p16) + cases: 90.7% (84.1-95.3) (ref: Alemany et al, Eur Urology, 2016)

HPV: human papillomavirus; CI: confidence interval

**F Head and neck cancers**

| **Country** | **Sex** | **N of new cancers irrespective of HPV status (95% CI)** | **N of new cancers attributable to HPV (mRNA and/or p16 positive), (95% CI)^11^** | **N of new cancers attributable to HPV 6/11/16/18/31/33/45/52/58 (mRNA and/or p16 positive), (95% CI)^12^** |
| --- | --- | --- | --- | --- |
| Austria | Women | 317 (282 - 352) | 27 (17 - 37) | 25 (15 - 35) |
|  | Men | 1107 (1042 - 1172) | 85 (67 - 103) | 80 (62 - 98) |
|  | Both sexes | 1424 (1350 - 1498) | 112 (91 - 133) | 105 (85 - 125) |
| Belgium | Women | 496 (452 - 540) | 39 (27 - 51) | 36 (24 - 48) |
|  | Men | 1957 (1870 - 2044) | 135 (112 - 158) | 127 (105 - 149) |
|  | Both sexes | 2453 (2356 - 2550) | 173 (147 - 199) | 163 (138 - 188) |
| Bulgaria | Women | 131 (109 - 153) | 11 (5 - 20) | 10 (5 - 18) |
|  | Men | 1050 (986 - 1114) | 59 (44 - 74) | 55 (40 - 70) |
|  | Both sexes | 1182 (1115 - 1249) | 70 (54 - 86) | 65 (49 - 81) |
| Croatia | Women | 124 (102 - 146) | 9 (4 - 17) | 8 (3 - 16) |
|  | Men | 897 (838 - 956) | 56 (41 - 71) | 53 (39 - 67) |
|  | Both sexes | 1021 (958 - 1084) | 65 (49 - 81) | 62 (47 - 77) |
| Cyprus | Women | 10 (5 - 18) | 1 (0 - 6) | 1 (0 - 6) |
|  | Men | 39 (27 - 51) | 2 (0 - 7) | 2 (0 - 7) |
|  | Both sexes | 49 (35 - 63) | 3 (1 - 9) | 2 (0 - 7) |
| Czech Republic | Women | 291 (258 - 324) | 27 (17 - 37) | 25 (15 - 35) |
|  | Men | 1464 (1389 - 1539) | 112 (91 - 133) | 106 (86 - 126) |
|  | Both sexes | 1755 (1673 - 1837) | 139 (116 - 162) | 132 (109 - 155) |
| Denmark | Women | 276 (243 - 309) | 21 (12 - 30) | 20 (11 - 29) |
|  | Men | 735 (682 - 788) | 53 (39 - 67) | 51 (37 - 65) |
|  | Both sexes | 1011 (949 - 1073) | 74 (57 - 91) | 70 (54 - 86) |
| Estonia | Women | 29 (18 - 40) | 2 (0 - 7) | 2 (0 - 7) |
|  | Men | 160 (135 - 185) | 10 (5 - 18) | 9 (4 - 17) |
|  | Both sexes | 189 (162 - 216) | 12 (6 - 21) | 11 (5 - 20) |
| Finland | Women | 169 (144 - 194) | 10 (5 - 18) | 10 (5 - 18) |
|  | Men | 350 (313 - 387) | 21 (12 - 30) | 20 (11 - 29) |
|  | Both sexes | 519 (474 - 564) | 32 (21 - 43) | 30 (19 - 41) |
| France | Women | 2614 (2514 - 2714) | 227 (197 - 257) | 214 (185 - 243) |
|  | Men | 14766 (14528 - 15004) | 1218 (1150 - 1286) | 1154 (1087 - 1221) |
|  | Both sexes | 17379 (17121 - 17637) | 1445 (1370 - 1520) | 1369 (1296 - 1442) |
| Germany | Women | 3429 (3314 - 3544) | 301 (267 - 335) | 284 (251 - 317) |
|  | Men | 13707 (13478 - 13936) | 1105 (1040 - 1170) | 1049 (986 - 1112) |
|  | Both sexes | 17136 (16879 - 17393) | 1406 (1333 - 1479) | 1334 (1262 - 1406) |
| Greece | Women | 212 (183 - 241) | 17 (10 - 27) | 16 (9 - 26) |
|  | Men | 1448 (1373 - 1523) | 83 (65 - 101) | 78 (61 - 95) |
|  | Both sexes | 1660 (1580 - 1740) | 101 (81 - 121) | 93 (74 - 112) |
| Hungary | Women | 294 (260 - 328) | 23 (14 - 32) | 22 (13 - 31) |
|  | Men | 1899 (1814 - 1984) | 137 (114 - 160) | 130 (108 - 152) |
|  | Both sexes | 2193 (2101 - 2285) | 160 (135 - 185) | 152 (128 - 176) |
| Iceland | Women | 8 (3 - 16) | 1 (0 - 6) | 1 (0 - 6) |
|  | Men | 16 (9 - 26) | 1 (0 - 6) | 1 (0 - 6) |
|  | Both sexes | 24 (14 - 34) | 1 (0 - 6) | 1 (0 - 6) |
| Ireland | Women | 122 (100 - 144) | 8 (3 - 16) | 7 (3 - 14) |
|  | Men | 367 (329 - 405) | 25 (15 - 35) | 23 (14 - 32) |
|  | Both sexes | 489 (446 - 532) | 33 (22 - 44) | 30 (19 - 41) |
| Italy | Women | 2334 (2239 - 2429) | 160 (135 - 185) | 147 (123 - 171) |
|  | Men | 9326 (9137 - 9515) | 558 (512 - 604) | 516 (471 - 561) |
|  | Both sexes | 11660 (11448 - 11872) | 718 (665 - 771) | 662 (612 - 712) |
| Latvia | Women | 37 (25 - 49) | 3 (1 - 9) | 2 (0 - 7) |
|  | Men | 265 (233 - 297) | 16 (9 - 26) | 15 (8 - 25) |
|  | Both sexes | 302 (268 - 336) | 18 (11 - 28) | 17 (10 - 27) |
| Lithuania | Women | 51 (37 - 65) | 4 (1 - 10) | 4 (1 - 10) |
|  | Men | 454 (412 - 496) | 31 (20 - 42) | 29 (18 - 40) |
|  | Both sexes | 504 (460 - 548) | 35 (23 - 47) | 33 (22 - 44) |
| Luxem-bourg | Women | 19 (11 - 30) | 2 (0 - 7) | 2 (0 - 7) |
|  | Men | 92 (73 - 111) | 8 (3 - 16) | 7 (3 - 14) |
|  | Both sexes | 110 (89 - 131) | 9 (4 - 17) | 9 (4 - 17) |
| Malta | Women | 11 (5 - 20) | 1 (0 - 6) | 1 (0 - 6) |
|  | Men | 46 (33 - 59) | 3 (1 - 9) | 3 (1 - 9) |
|  | Both sexes | 58 (43 - 73) | 4 (1 - 10) | 4 (1 - 10) |
| Norway | Women | 139 (116 - 162) | 10 (5 - 18) | 10 (5 - 18) |
|  | Men | 359 (322 - 396) | 27 (17 - 37) | 26 (16 - 36) |
|  | Both sexes | 498 (454 - 542) | 37 (25 - 49) | 35 (23 - 47) |
| Poland | Women | 925 (865 - 985) | 66 (50 - 82) | 61 (46 - 76) |
|  | Men | 4825 (4689 - 4961) | 277 (244 - 310) | 257 (226 - 288) |
|  | Both sexes | 5749 (5600 - 5898) | 343 (307 - 379) | 318 (283 - 353) |
| Portugal | Women | 285 (252 - 318) | 19 (11 - 30) | 16 (9 - 26) |
|  | Men | 3103 (2994 - 3212) | 216 (187 - 245) | 202 (174 - 230) |
|  | Both sexes | 3388 (3274 - 3502) | 236 (206 - 266) | 219 (190 - 248) |
| Romania | Women | 388 (349 - 427) | 31 (20 - 42) | 29 (18 - 40) |
|  | Men | 3328 (3215 - 3441) | 225 (196 - 254) | 212 (183 - 241) |
|  | Both sexes | 3716 (3597 - 3835) | 256 (225 - 287) | 240 (210 - 270) |
| Slovakia | Women | 121 (99 - 143) | 10 (5 - 18) | 9 (4 - 17) |
|  | Men | 1175 (1108 - 1242) | 93 (74 - 112) | 88 (70 - 106) |
|  | Both sexes | 1296 (1225 - 1367) | 102 (82 - 122) | 97 (78 - 116) |
| Slovenia | Women | 58 (43 - 73) | 5 (2 - 12) | 4 (1 - 10) |
|  | Men | 363 (326 - 400) | 27 (17 - 37) | 26 (16 - 36) |
|  | Both sexes | 422 (382 - 462) | 32 (21 - 43) | 30 (19 - 41) |
| Spain | Women | 1443 (1369 - 1517) | 91 (72 - 110) | 83 (65 - 101) |
|  | Men | 9092 (8905 - 9279) | 550 (504 - 596) | 509 (465 - 553) |
|  | Both sexes | 10534 (10333 - 10735) | 642 (592 - 692) | 592 (544 - 640) |
| Sweden | Women | 281 (248 - 314) | 20 (11 - 29) | 19 (11 - 30) |
|  | Men | 609 (561 - 657) | 49 (35 - 63) | 46 (33 - 59) |
|  | Both sexes | 890 (832 - 948) | 69 (53 - 85) | 66 (50 - 82) |
| Switzer-land | Women | 357 (320 - 394) | 31 (20 - 42) | 29 (18 - 40) |
|  | Men | 1172 (1105 - 1239) | 93 (74 - 112) | 88 (70 - 106) |
|  | Both sexes | 1529 (1452 - 1606) | 124 (102 - 146) | 118 (97 - 139) |
| The Nether-lands | Women | 741 (688 - 794) | 52 (38 - 66) | 49 (35 - 63) |
|  | Men | 1828 (1744 - 1912) | 115 (94 - 136) | 108 (88 - 128) |
|  | Both sexes | 2569 (2470 - 2668) | 166 (141 - 191) | 157 (132 - 182) |
| United Kingdom | Women | 2341 (2246 - 2436) | 168 (143 - 193) | 156 (132 - 180) |
|  | Men | 5991 (5839 - 6143) | 444 (403 - 485) | 415 (375 - 455) |
|  | Both sexes | 8331 (8152 - 8510) | 612 (564 - 660) | 571 (524 - 618) |

^11^ HPV DNA and (mRNA or p16) prevalence: 3.7% (95% CI: 2.4-5.6) in oral cavity cancers, 10.8% (95% CI: 3.0-25.4) in nasopharyngeal cancers, 19.9% (95% CI: 17.2-22.8) in oropharyngeal cancers, 2.4% (95% CI: 0.3-8.4) in hypopharyngeal cancers, 25.0% (95% CI: 10.7-44.9) in pharyngeal cancers and 2.4% (95% CI: 1.2-4.1) in laryngeal cancers ^12^ HPV DNA and (mRNA or p16) 6/11/18/31/33/45/52/58 attributable fraction among HPV DNA and (mRNA or p16) + cases: 90.9% (95% CI: 70.8-98.9) in oral cavity cancers, 75.0% (95% CI: 19.4-99.4) in nasopharyngeal cancers, 97.5% (95%CI: 93.7-99.3) in oropharyngeal cancers , 100% (95%CI: 15.8-100) in hypopharyngeal cancers, 85.7% (95% CI: 42.1-99.6) in pharyngeal cancers and 91.7% (95% CI: 61.5-99.8) in laryngeal cancers (ref: Castellsagué et al, J Natl. cancer Inst., 2016)

HPV: human papillomavirus; CI: confidence interval.
